# A new approach for location-specific seasonal outlooks of typhoon and super typhoon frequency across the Western North Pacific region

**DOI:** 10.1038/s41598-021-98329-6

**Published:** 2021-09-30

**Authors:** Andrew D. Magee, Anthony S. Kiem, Johnny C. L. Chan

**Affiliations:** 1 Centre for Water, Climate and Land (CWCL), University of Newcastle, Callaghan, Australia; 2 School of Energy and Environment, City University of Hong Kong, Hong Kong, China

**Keywords:** Climate sciences, Natural hazards

## Abstract

With an average of 26 tropical cyclones (TCs) per year, the western North Pacific (WNP) is the most active TC basin in the world. Considerable exposure lies in the coastal regions of the WNP, which extends from Japan in the north to the Philippines in the south, amplifying TC related impacts, including loss of life and damage to property, infrastructure and environment. This study presents a new location-specific typhoon (TY) and super typhoon (STY) outlook for the WNP basin and subregions, including China, Hong Kong, Japan, Korea, Philippines, Thailand, and Vietnam. Using multivariate Poisson regression and considering up to five modes of ocean-atmospheric variability and teleconnection patterns that influence WNP TC behaviour, thousands of possible predictor model combinations are compared using an automated variable selection procedure. For each location, skillful TY and STY outlooks are generated up to 6 months before the start of the typhoon season, with rolling monthly updates enabling refinement of predicted TY and STY frequency. This unparalleled lead time allows end-users to make more informed decisions before and during the typhoon season.

## Introduction

The western North Pacific (WNP; 0°–60ׄ° N, 100° E–180°) is the most active tropical cyclone (TC) basin in the world, with an average of approximately 26 TCs (including tropical storms, typhoons (TYs) and super typhoons (STYs)) per year (1981–2010)^1^. TCs bring strong winds, prolonged and intense rainfall, and storm surge and cause significant loss of life and substantial damage to property, infrastructure and the environment due to the immense exposure that exists around the coastal fringe of the WNP basin^2^. Although WNP TCs are a regular occurrence, they wreak havoc and have devastating economic, social and environmental impacts^3,4^. Temporally and spatially, TCs are erratic and no two seasons are the same^4^. For example, 30 named TCs occurred over the WNP in 2004, ten of which made landfall across Japan (breaking the previously held record of 6), while in 2010, only 14 named TCs formed in the basin, two of which made landfall across Japan^1^.

Outlooks that predict WNP TC frequency play an essential role in bridging the gap between extreme weather forecasting (lead times of days to weeks before an event) and the need for governments, decision-makers, aid agencies, insurers/reinsurers and many others to make longer-term decisions months before the start of the TY season. Outlooks are possible because the seasonal predictability of TCs are mainly controlled by slowly evolving external forcing, including changes in sea surface temperatures (SSTs) and large-scale atmospheric circulation patterns^1^. Many TC prediction schemes for the WNP and or individual countries within the WNP region exist, including statistical^5–11^, dynamical^12–14^ and hybrid statistical–dynamical outlooks^15–18^, all of which use one or several external forces known to influence TC activity as model predictors.

Intraseasonal, interannual and interdecadal climate variability drives changes in TC behaviour, including cyclogenesis and track morphology across the WNP region^13,19–25^. El Niño-Southern Oscillation (ENSO), the dominant mode of tropical interannual variability, is relatively well understood and is known to displace TCs significantly towards the east in the developing year of an El Niño phase^19–23^. El Niño seasons also result in more frequent and intense TC events^22^ and drive increased incidence of northward recurving TCs towards the extratropics resulting in more landfalling TCs for Japan and Korea. More recently, the emergence of ENSO-related diversity^26^, e.g. Central Pacific El Niño (Modoki)^27^, is comparatively less understood but influences the response of basin-wide WNP TC activity^23^, with studies indicating a west/northwest shift in TY activity, with more landfalling TC events across East Asia^24^. With ENSO Modoki events becoming increasingly common, the consideration of ENSO diversity is essential in modelling efforts^28,29^.

Indian Ocean SST variability, particularly warm (cool) SST anomalies (SSTAs) in the eastern Indian Ocean (EIO), are found to result in more (less) TCs in the WNP^13,25^. EIO SSTAs are known to influence TC genesis frequency in two ways. First, cool (warm) EIO SSTAs promote enhanced (reduced) land-sea thermal contrast, resulting in a stronger (weaker) than normal East Asian and WNP summer monsoon, promoting (suppressing) TC genesis over the WNP. Second, cool (warm) EIO SSTAs can cause a cold (warm) equatorial Kelvin wave over the WNP region resulting in more (less) favourable conditions required for TC genesis^13,22^. While both ENSO and Indian Ocean SST variability, particularly EIO SSTAs have a significant influence on TC behaviour in the WNP, their effects are quite different, and consideration of both ENSO and EIO SSTAs could improve seasonal prediction of WNP TC activity^22^.

The seasonal predictability of TCs may be assisted by including other lower frequency modes of atmospheric variability and/or teleconnection patterns^1^. The Pacific/North American Pattern (PNA) is inversely correlated with WNP TC frequency, where low (high) PNA years result in more (less) favourable large-scale dynamic conditions required for TC genesis. Considering variations in the PNA pattern is recommended as this may improve the predictive skill of WNP TC frequency^30^. The westerly phase of the Quasi-Biennial Oscillation (QBO)^31–33^ favours cyclogenesis across the WNP through more favourable low-level relative vorticity, high-level divergence, tropospheric vertical wind shear and mid-level humidity^1,32,34^. Lastly, a positive (negative) Pacific Meridional Mode (PMM) is also found to result in more (less) TCs occurring across the WNP primarily driven by changes in zonal vertical wind shear^35–38^. Incorporating multiple ocean, atmospheric and coupled ocean-atmospheric modes of variability and teleconnection patterns into a predictive modelling framework has the potential to derive more robust and skillful TY and STY forecasts for the WNP.

In this study, we apply a statistical modelling framework (multivariate Poisson regression) to generate location-specific TY (maximum 10-minute sustained winds ≥ 64 kt and < 114 kt) and STY (maximum 10-minute sustained winds ≥ 114 kt) outlooks for the WNP, China, Hong Kong, Japan, Korea, Philippines, Thailand and Vietnam. Following the recommendations of ^39^, location-specific outlooks provide more useful and bespoke guidance for individual countries/regions. Our models consider ENSO diversity by including ten oceanic, atmospheric and coupled ocean–atmosphere ENSO indices alongside other influences (Indian Ocean SST variability, PNA, QBO and the PMM) that drive changes in TC behaviour across the WNP (Fig. S1). Models are initialised, trained and validated every month for 9 monthly lead-times (six pre-season outlooks; up to 6 months before the start of the season) and three in-season outlooks. For each lead time and location, thousands of possible predictor model combinations are tested, and the most skillful combination is selected for further analysis. The benefits of deriving skillful TY and STY outlooks up to 6 months before the start of the TY season and providing rolling monthly updates before and during the season are widespread^28,29^. While TCs are a regular occurrence and financial loss and death will continue to be a reality well into the future, skillful and location-specific TC outlooks generated months before the start of the typhoon season can inform and improve decision-making aimed at reducing the impacts associated with these extreme events.

## Results

### Defining the TY season

**Figure 1:**
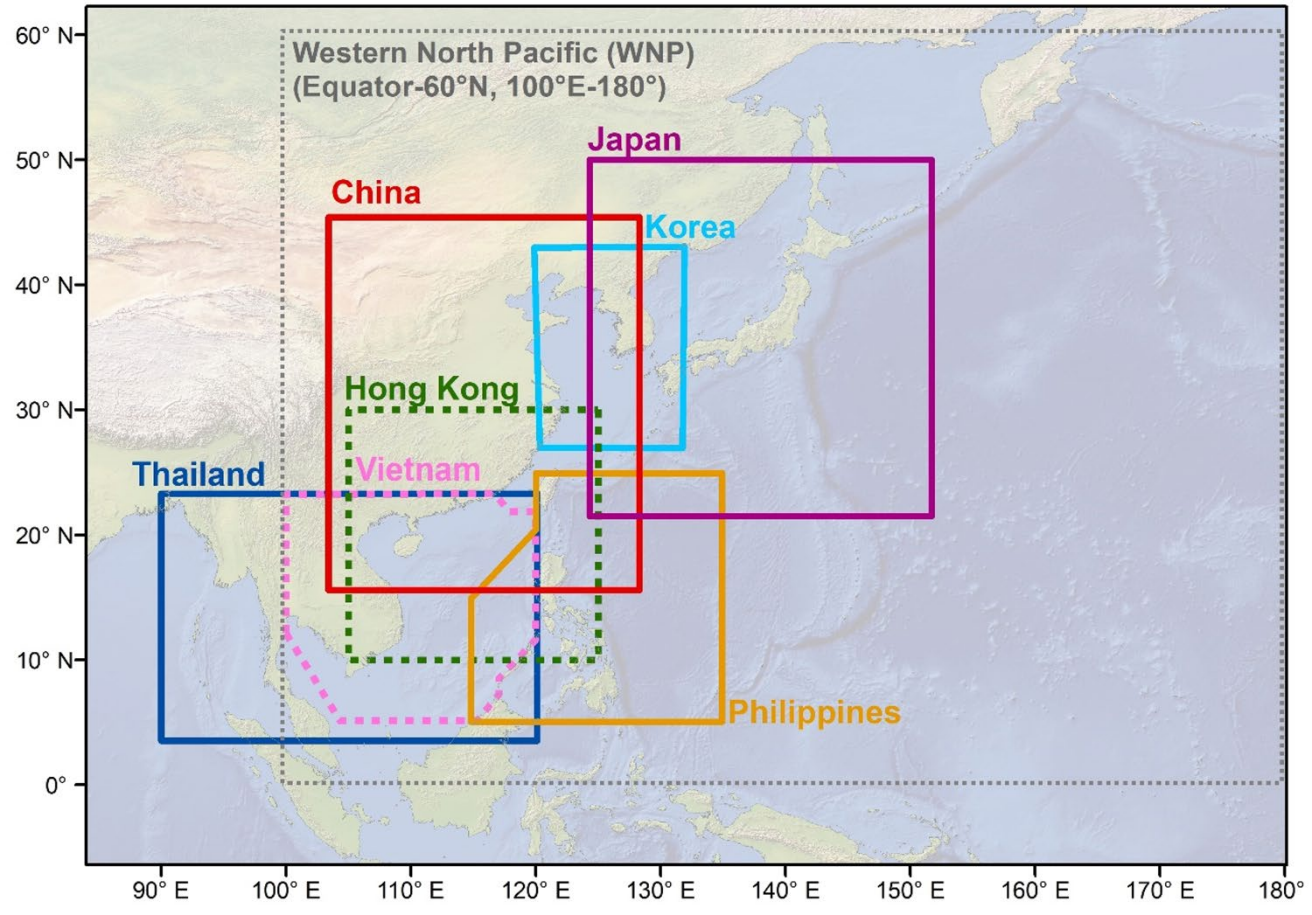
Western North Pacific (WNP) and seven sub-regional locations considered in this analysis. Figure created using a baseamp from Natural Earth (www.naturalearthdata.com).

**Figure 2:**
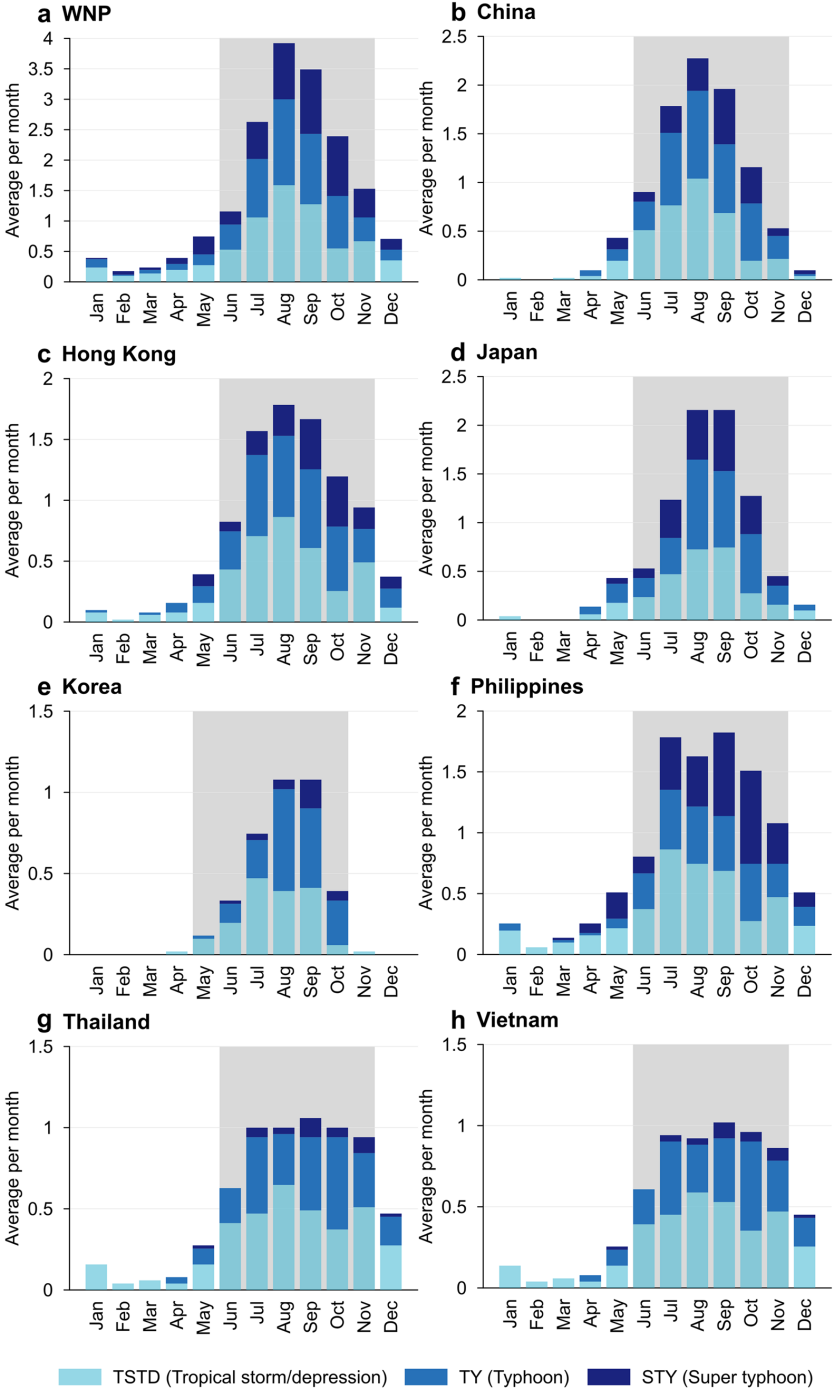
Monthly climatology (average per month) of western North Pacific (WNP) tropical storm/depressions (TSTD), typhoons (TY), and super typhoons (STY) between 1987 and 2020. Grey shaded area indicates most active 6-month period for each location and defines the 6-month typhoon season modelled in this analysis.

The monthly climatology of TSTD, TY and STY events (1987–2020) is calculated for the WNP and seven individual countries/regions, including China, Hong Kong, Japan, Korea, Philippines, Thailand and Vietnam (Fig. 1). The subjective nature used to define the TY season across the WNP and the geographical spread of countries considered in this analysis means that TY seasons can differ between agencies. To account for this objectively, the most active 6-month period is used to define the TY season for each region (Fig. 2). Doing so revealed two different TY seasons: June–November (WNP, China, Hong Kong, Japan, Philippines, Thailand and Vietnam) and May–October (Korea). While TCs can occur in any month of the year, the defined TY seasons using the described methodology account for between 78% (Philippines) and 99% (Korea) of TSTD events, between 88% (Philippines) and 100% (Korea) of TY events and between 86% (WNP) and 100% (Korea) of STY events.

### Comparing model skill for TY and STY outlooks

In this study, we evaluated the performance of 10 unique predictor models in producing skillful TC outlooks, each of which pairs a unique ENSO index with other ocean/atmospheric climate influences that drive changes to WNP TC behaviour (see Table S1)^19–23^. These indices include the Dipole Mode Index (DMI)^40^, including the Indian Ocean Dipole East Box (IOD E) and West Box (IOD W)^40^, the Pacific/North American Pattern (PNA)^41^, Quasi-biennial Oscillation (QBO)^31^ and the Pacific Meridional Mode (PMM)^42^ (see “Data and model development” and Table 1). An automated variable selection procedure is applied to select the optimum combination of predictors for each of the ten predictor models (Table S1) using a generalised linear model with a Poisson distribution and log link function to predict the mean number of TCs per season. After generating a training time series and a Leave-One Out Cross-Validation (LOOCV) time series, the model that generates the highest skill score (SS) for the LOOCV series is selected for further analysis. Values from the LOOCV analysis are presented throughout the manuscript and values from the training analysis are presented in the “Supplementary Material”.

**Table 1:** Model covariates used to build predictor models, including ENSO indices (1–10) and non-ENSO indices (11–16).

	Index	Detail	Source	References
1	NINO1 + 2	Average SSTs in the NINO1 + 2 region (0°–10° S, 90°–80° W)	ERSSTv5^43^	^44^
2	NINO3	Average SSTs in the NINO3 region (5° N–5° S, 150°–90° W)		
3	NINO3.4	Average SSTs in the NINO3.4 region (5°–5° S, 170°–120° W)		^45^
4	NINO4	Average SSTs in the NINO4 region (5° N–5° S, 160° E–150° W)		^46^
5	Southern Oscillation Index (SOI)	Atmospheric index calculated using the pressure differences between Tahiti and Darwin	NOAA CPC	^47^
6	Coupled ENSO Index (CEI)	Three-month smoothed NINO3.4 and SOI using a 1970–2018 anomaly period	ERSSTv5 for NINO3.4 and NOAA CPC for SOI	^48^
7	Oceanic NINO Index (ONI)	Three-month running mean of NINO3.4 SSTs, based on changing base period consisting of sliding centred 30-year base periods	ERSSTv5^43^	^49^
8	Trans NINO Index (TNI)	Difference in normalised SST anomalies between the NINO1 + 2 and NINO4 regions	HadSST1.1^50^ until Nov 1981 and NCEP NOAA OI^51^ after	^44^
9	ENSO Modoki Index (EMI)	Difference in monthly SSTs between Modoki A (10° N–10° S, 165° E–140° W), Modoki B (5° N–15° S, 110°–70° W), and Modoki C (20° N–10° S, 125°–145° E) and calculated using the following equation: *EMI* = *Modoki A* − (0.5*Modoki B) − (0.5*Modoki C)	ERSSTv5^43^	^24^
10	ENSO Longitudinal Index (ELI)	Index based on the longitudinal extent of ENSO	^52^	^52^
11	Indian Ocean Dipole (IOD) East box (IOD E)	SST anomalies in the IOD E region (eastern pole of DMI; 0°–10° S, 90°–110° E)	ERSSTv5^43^	^40^
12	Indian Ocean Dipole (IOD) West box (IOD W)	SST anomalies in the IOD W region (western pole of the DMI; 10° N–10° S, 50°–70° E)	ERSSTv5^43^	
13	Dipole Mode Index (DMI)	Difference in SST anomalies between IOD W and IOD E	ERSSTv5^43^	
14	Pacific/North American Pattern (PNA)	Rotated Principal Component Analysis (RPCA) applied to monthly standardised 500mb height anomalies between 20° and 90°N	NOAA CPC	^41^
15	Quasi-Biennial Oscillation (QBO)	Zonal average of the equatorial 30mb zonal wind	NOAA PSL	^31^
16	SST Pacific Meridional Mode (PMM)	Maximum Covariance Analysis (MCA) to SSTs (21° S–32° N, 74° W–15° E)	NCEP SST	^42^

**Figure 3:**
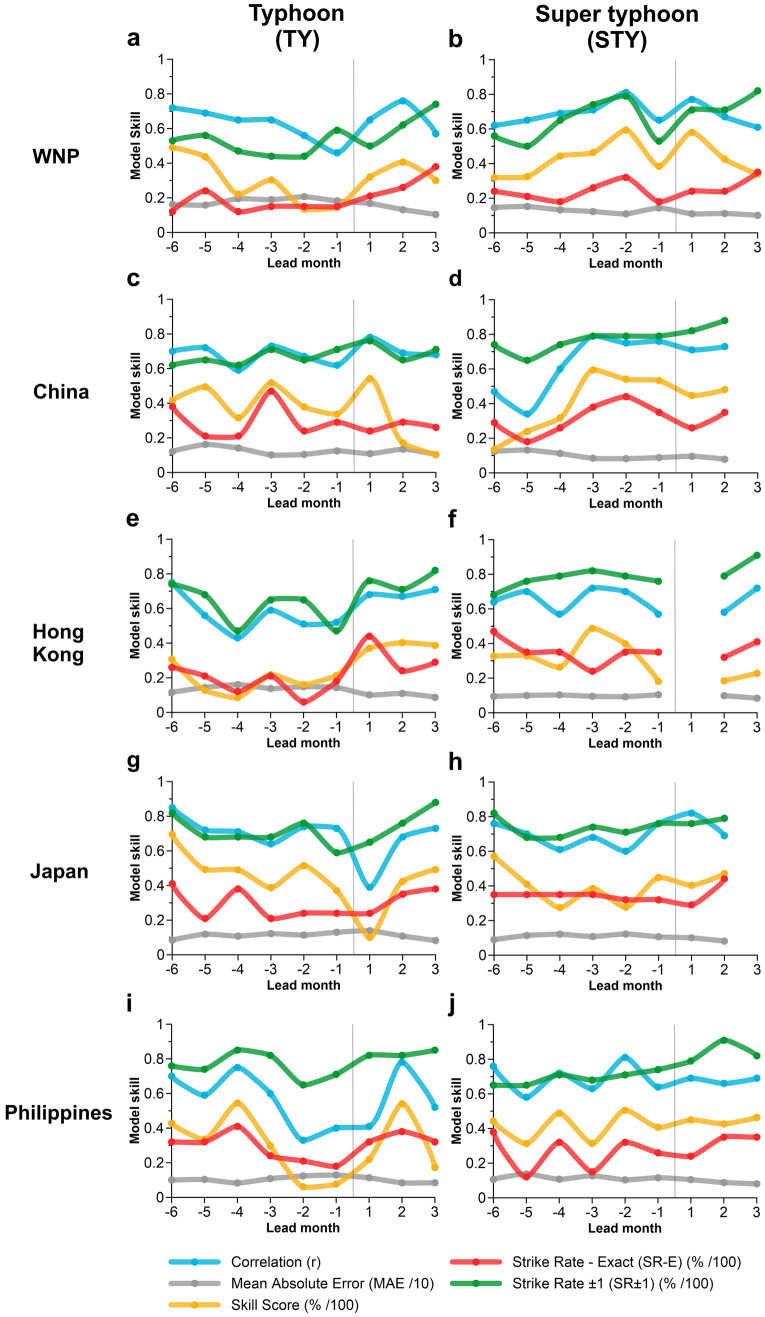
Evaluation of Leave-One-Out Cross-Validation (LOOCV) model performance for model lead times (leads − 6 to + 3) for TYs (left panels) and STYs (right panels) for the WNP (**a**, **b**), China (**c**, **d**), Hong Kong (**e**, **f**), Japan (**g**, **h**) and the Philippines (**i**, **j**) for each respective 6 month typhoon season between 1987 and 2020. The vertical line separates the pre-season (lead − 1 to lead − 6) and in-season (lead + 1 to lead + 3) outlooks. Lead months with missing model performance statistics (e.g. lead + 1 in **f**) indicates negligible/no model skill.

The skill of TY (maximum sustained winds ≥ 64 kt and < 114 kt) and STY (maximum sustained winds ≥ 114 kt) outlooks for a range of model initialisation periods (six pre-season and three in-season outlooks) are evaluated in Fig. 3. Individual TY and STY outlooks are only presented for the WNP, China, Hong Kong, Japan and the Philippines as other countries (Korea, Thailand and Vietnam) had negligible/no skill due to insufficient TY and STY numbers. However, outlook skill for all regions where maximum sustained winds ≥ 64 kt, including both TY and STY events (herein TY + STY) is assessed in the following section.

For TYs, skillful predictions can be made up to 6 months (December) before the start of the TY season (June) (Fig. 3, left panels). LOOCV model statistics suggest that at lead − 6 (the preceding December), pre-season outlooks are most skillful for the WNP, with a skill score (SS) of 48%, correlation between the LOOCV predicted and the observed TY time series of r = 0.72 (significant at p < 0.01), an exact strike rate (SR-E; where the LOOCV prediction, rounded to the nearest count, exactly matches the observation) of 12% (4 seasons of 34 TY seasons) and an SR ± 1 (where the LOOCV outlook matches the observation ± 1 TY) of 53% (18 seasons of 34 TY seasons). Lead − 6 pre-season outlooks are also most skillful for Japan (SS = 69% and SR-E = 41%) and Hong Kong, (SS = 31% and SR-E = 26%). For the other locations, lead − 3 is the most skillful model initialisation period for China (SS = 52% and SR-E = 47%) and lead − 4 is most skillful for the Philippines (SS = 54% and SR-E = 41%). Interestingly, for the WNP, Japan and the Philippines, the SS typically decreases with reduced lead time, indicating that these models are able to detect important pre-conditioning of parameters most suited to TC formation and movement.

Compared to TYs, the most skillful pre-season STY outlooks are typically generated closer to the start of the season, perhaps due to fewer STYs than TY counts (Fig. 3; right panels). Outlooks generated at lead − 2 (April) are most skillful for the WNP (SS = 59% and SR-E = 32%) and the Philippines (SS = 51% and SR-E = 32%) and outlooks generated at lead − 3 (March) are most skillful for China (SS = 59% and SR-E = 38%) and Hong Kong (SS = 49% and SR-E = 24%). However, for Japan, outlooks generated at lead − 6 (the preceding December) are more skillful than any other lead month (SS = 57% and SR-E = 82%). Regardless for other locations, useful information about STY frequency can still be obtained by generating the outlook up to lead − 6 where the SS ranges from between 13% (China) up to 57% (Japan) and SR-E ranges from between 24% (WNP) up to 47% (Hong Kong).

While pre-season outlooks provide information about the fixed 6-month typhoon season across six lead times, in-season outlooks provide valuable information about TY and STY frequency for the remaining season e.g. models initialised between lead months + 1 to + 3 provide guidance for the remaining 5–3 months of the season, respectively. This enables greater temporal granularity of when TYs and STYs may occur, but at the detriment of lead time. This is particularly useful for locations where the latter half of the season is more active than the first half (e.g. Philippines). However, given the length of the season (and thus TY and STY frequency) varies with increased in-season lead time, skill should not be compared. Generally, in-season TY and STY outlooks demonstrate good performance, demonstrating skill in both SS and SR-E metrics. For example, China (Philippines) TY outlooks have SS ranging between 10 and 54% (17–53%) and WNP (Philippines) STY outlooks have SS ranging from between 34 and 58% (43–46%).

**Figure 4:**
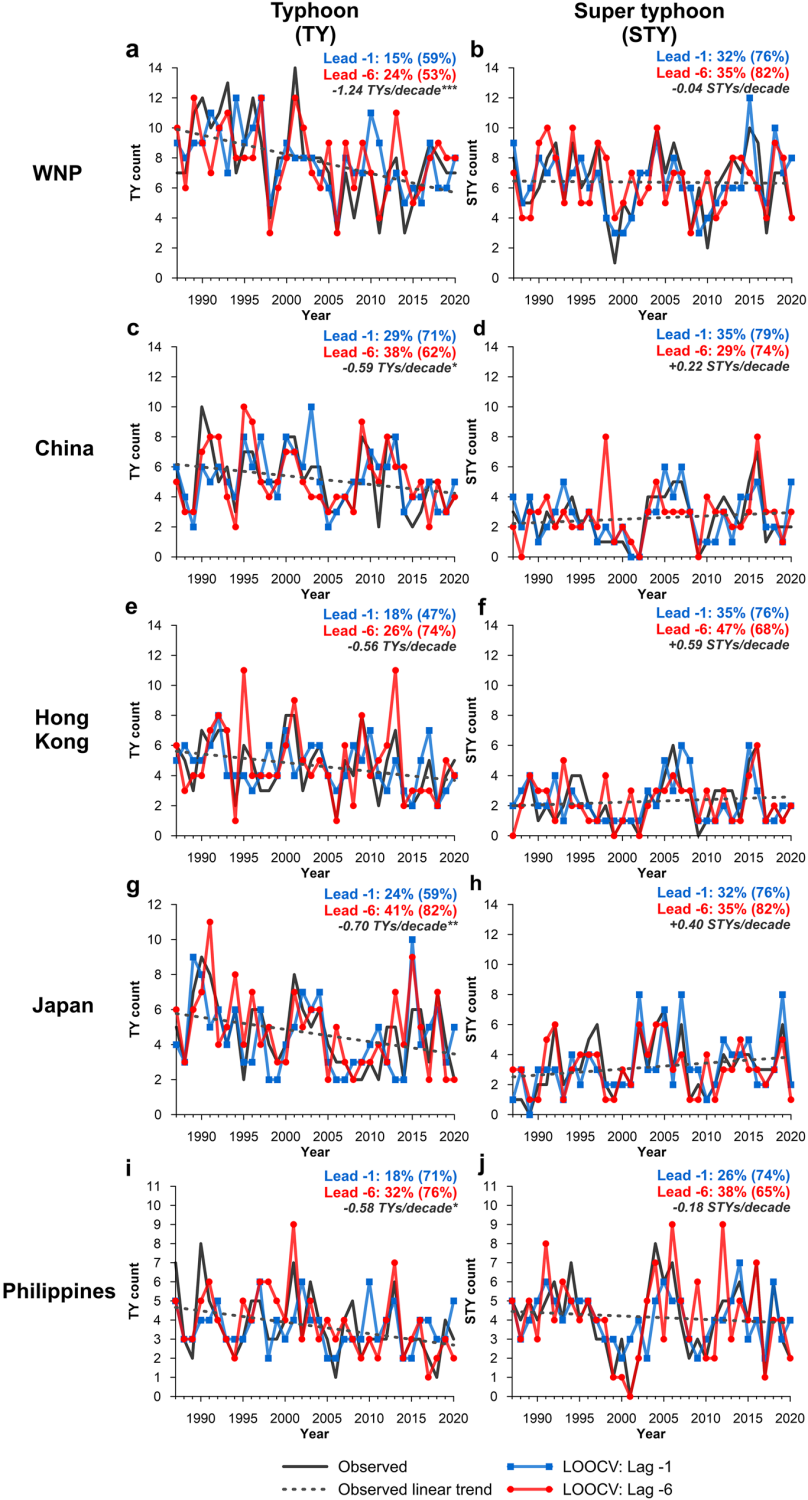
Comparison of observed and LOOCV predicted TYs (left column) and STYs (right column) for the WNP (**a**, **b**), China (**c**, **d**), Hong Kong (**e**, **f**), Japan (**g**, **h**) and the Philippines (**i**, **j**) between 1987 and 2020. The LOOCV prediction is compared for two pre-season periods: lead − 1 (1 month before the start of the typhoon season; blue line) and lead − 6 (6 months before the start of the typhoon season; red line). On-panel percentage values indicate LOOCV SR-E (SR ± 1 in parentheses) for models lead − 1 and lead − 6. Dashed line represents observed linear trend with on-panel trend (/decade) summarised in grey italics with statistical significance (Mann–Kendall test) denoted by an asterisk (*significant at 90% confidence level; **significant at 95% confidence level; ***significant at 99% confidence level).

Comparison of observed and LOOCV predicted TYs (Fig. 4; left panels) and STYs (Fig. 4; right panels) demonstrates the LOOCV models ability at lead − 1 and lead − 6 to capture the season-to-season variability of TY/STY counts and in terms of SR-E/SR ± 1, the superior model performance at lead − 6. However, there are some cases where predictions generated at lead − 6 tend to overestimate, e.g. the 1995 Hong Kong TY season, where lead − 6 (− 1) LOOCV predicted 11 (4) TYs compared to 6 TYs observed. Similar overestimation occurs for lead − 6 outlooks during the 1991 Japan TY season (11 TYs predicted compared to 8 TYs observed). Regardless, no seasonal TC outlook is skillful 100% of the time due to the multiple and competing influences that drive variability in TC behaviour. Importantly, the LOOCV time series presented is representative of model performance in ‘validation mode’ and the same overestimations are not observed in the training time series (Fig. S3). The LOOCV models are also able to capture the observed linear trends in TY and STYs. For TYs, significant downward trends (≥ 90% confidence level; Mann–Kendall Trend Test) are observed for the WNP (− 1.24 TYs/decade), China (− 0.59 TYs/decade), Japan (− 0.70 TYs/decade) and the Philippines (− 0.58 TYs/decade). For STYs, no significant trends were observed, but an increasing trend in STY frequency was observed for China (0.22 STYs/decade), Hong Kong (0.59 STYs/decade) and Japan (0.40 STYs/decade). Negligible non-significant trends in STY frequency were observed for the WNP (− 0.04 STYs/decade) and the Philippines (− 0.18 STYs/decade).

### Comparing model skill for all TYs

**Figure 5:**
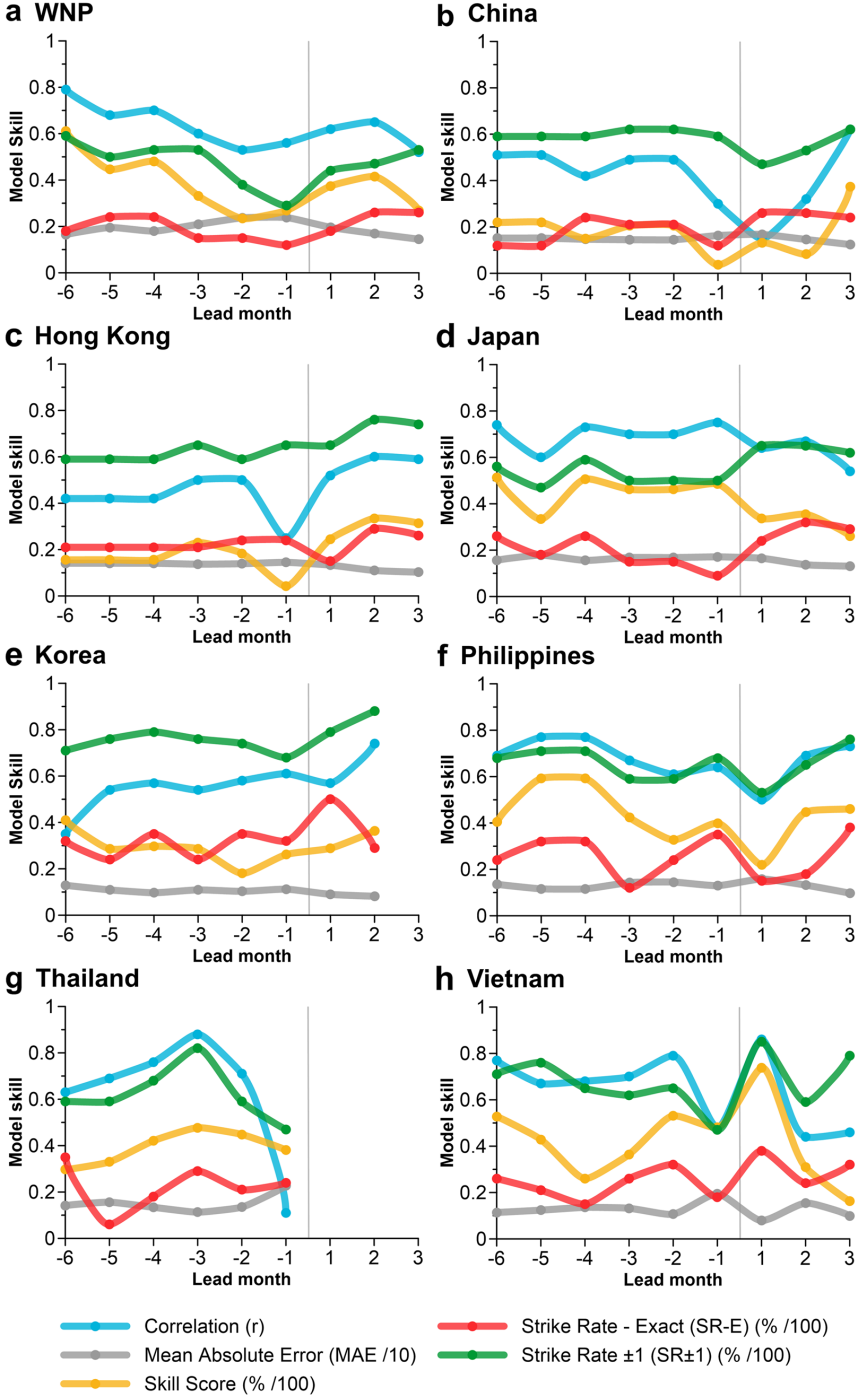
Evaluation of Leave-One-Out Cross Validation (LOOCV) model performance for all typhoons (where maximum sustained winds ≥ 64 kt; TY + STY events) for eight locations between 1987 and 2020. The vertical line separates the pre-season (lead − 1 to lead − 6) and in-season (lead + 1 to lead + 3) outlooks. Lead months with missing model performance statistics (e.g. Lead + 3 for **e**) indicates negligible/no model skill.

As individual TY and STY outlooks for Korea, Thailand and Vietnam resulted in negligible skill, TY + STY models are derived (where maximum sustained winds ≥ 64 kt) for the WNP and seven other locations considered in this analysis. Consistent with previous findings (Fig. 3), TY + STY outlooks generated at lead − 1 are typically not the most skillful (Fig. 5). Instead, outlooks generated between lead − 2 and lead − 6 are the most skillful. For the WNP, China, Hong Kong, Japan and the Philippines, TY + STY model skill is comparable to individual TY and STY outlooks (Fig. 3), so the remaining discussion will focus on Korea, Thailand and Vietnam. For Korea, pre-season outlooks generated at lead − 6 are most skillful (SS = 41%, SR-E = 32%) with a fairly consistent SS between lead − 5 and lead − 3. For Thailand, lead − 3 (March) is the most skillful model initialisation period (SS = 48%, SR-E = 29%), however a substantial decline in model skill for lead − 2 and lead − 1 is evident, and no skillful in-season outlooks are available. Lastly, for Vietnam, lead − 2 (April) is most skillful (SS = 53%, SR-E = 35%), with models initiated in lead − 6 (December) a close second (SS = 52%, SR-E = 26%). In-season model skill is also impressive; however, not all lead times produced skillful models (e.g. Thailand), likely due to a diminishing sample size.

**Figure 6:**
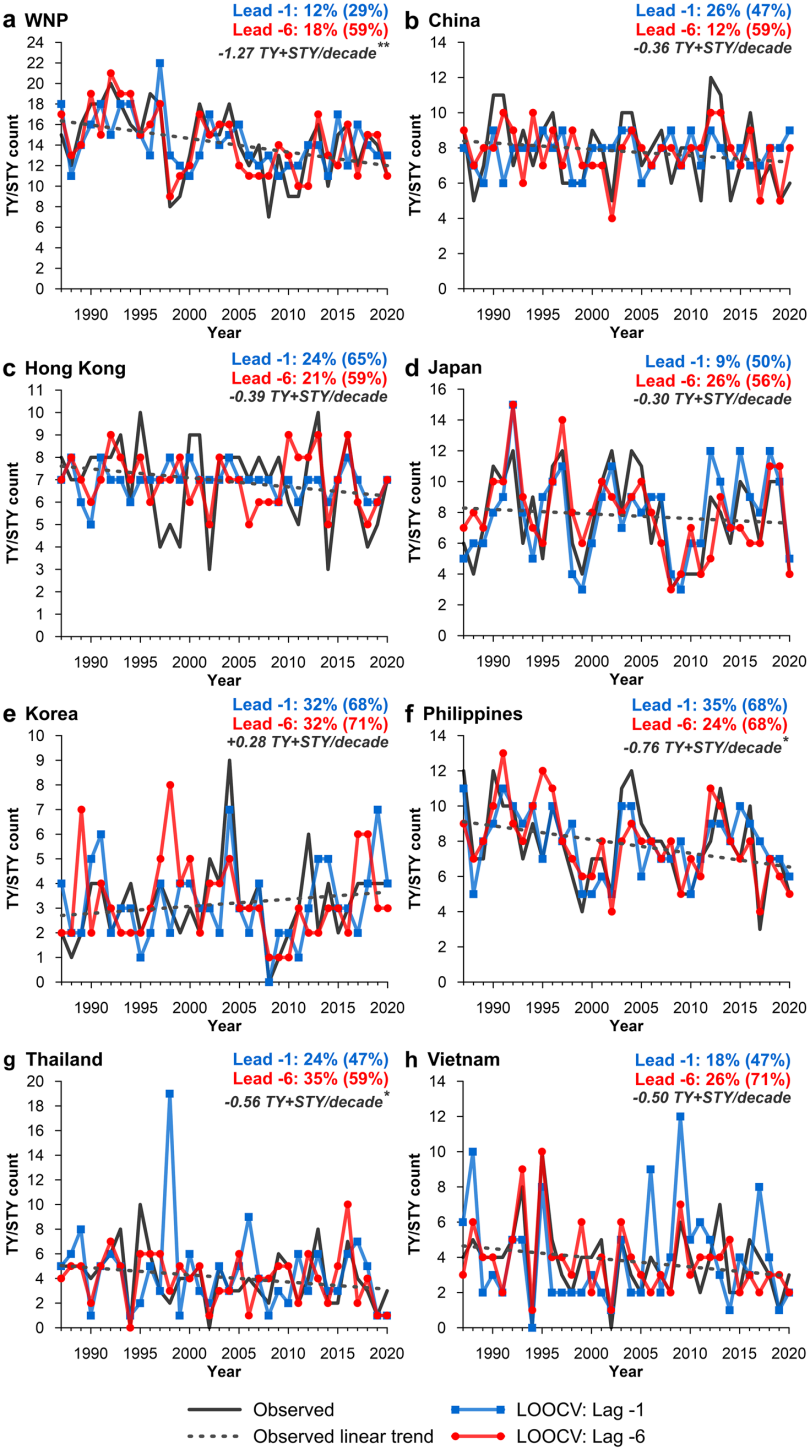
Comparison of observed and LOOCV predicted typhoons (TY and STY events) between 1987 and 2020. The LOOCV prediction is compared for two pre-season periods: lead − 1 (1 month before the start of the typhoon season; blue line) and lead − 6 (6 months before the start of the typhoon season; red line). On-panel percentage values indicate LOOCV SR-E (SR+ − 1 in parentheses) for models lead − 1 and lead − 6. Dashed line represents observed linear trend with on-panel trend (/decade) summarised in grey italics with statistical significance (Mann–Kendall test) denoted by an asterisk (*significant at 90% confidence level; **significant at 95% confidence level; ***significant at 99% confidence level).

Of the eight regions considered, seven have a decreasing trend in TY + STY counts, three of which are statistically significant (≥ 90% confidence level), including the WNP (− 1.27 TY + STY/decade), the Philippines (− 0.76 TY + STY/decade) and Thailand (− 0.56 TY + STY/decade) (Fig. 6). Korea is the only location to see an increasing trend in the number of TY + STY events, however, this trend is not statistically significant (+ 0.28 TY + STY/decade). Both lead − 1 and lead − 6 LOOCV time series’ capture the trends in observed TC counts well. Some overestimation is observed (e.g. 1998 season in Thailand) and is most typical in lead − 1 outlooks, unlike individual TY and STY outlooks where lead − 6 outlooks were more likely to overestimate (Fig. 4).

**Figure 7:**
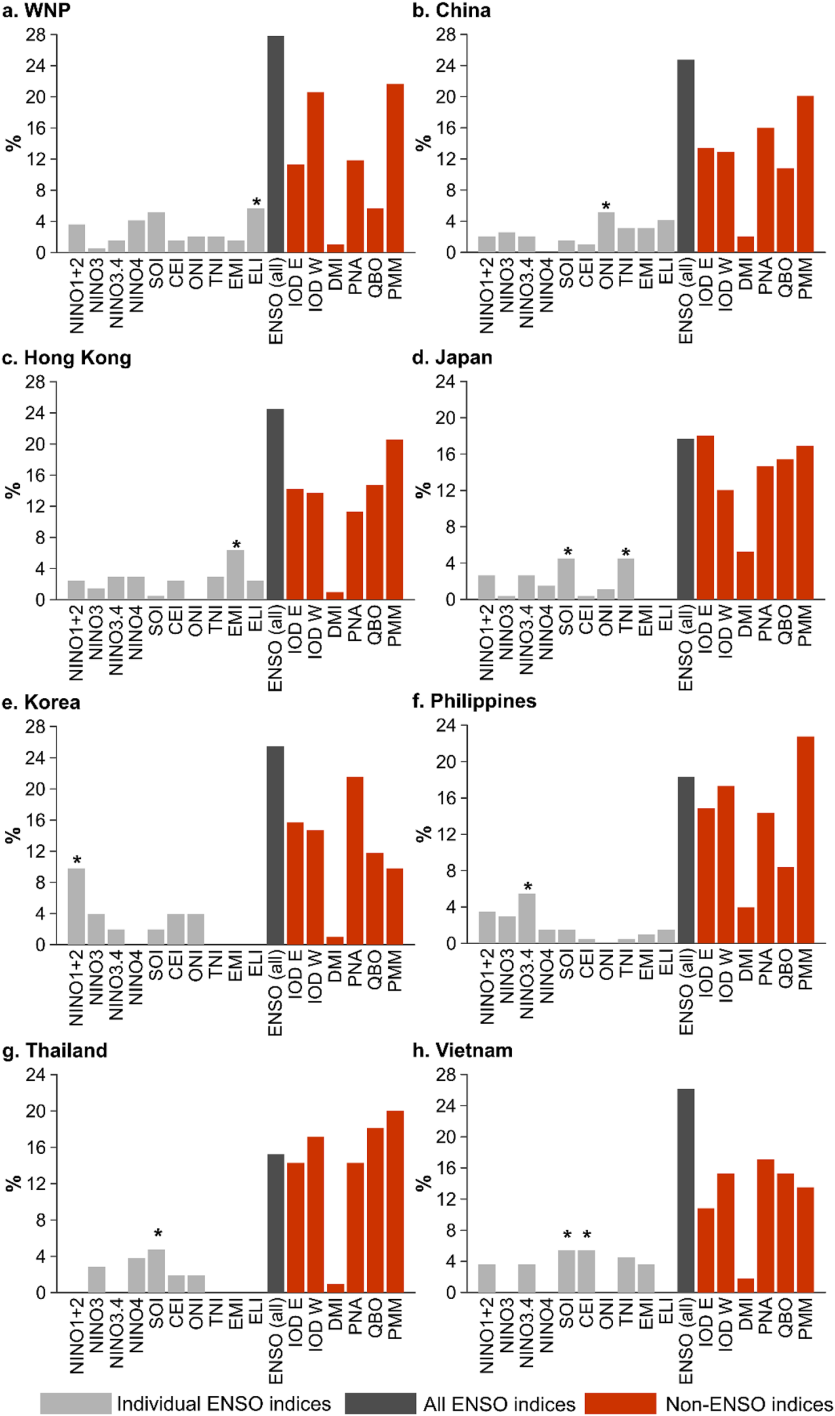
Proportion (%) of selected model covariates using the automated variable selection procedure for all outlook model runs (TY, STY and TY + STY) and nine model initialisation lead times. Light grey bars represent individual ENSO indices (note that the proportion of individual ENSO indices should not be compared with all ENSO indices (dark grey bar) and non-ENSO indices (red bars) as not all ENSO indices feature in each model run). All ENSO indices (dark grey bar) represents the aggregate proportion of individual ENSO indices and can be compared with non-ENSO indices (red bars). The asterisk indicates the most commonly selected ENSO index.

The stepAIC function selected, on average, nine covariates for TY, STY and TY + STY forecasts and eight covariates for STY forecasts; however models ranges from including as few as two covariates to as many as 19. For each model run, up to 42 covariates in total were available for selection. While parsimony varies between models, only models with AICc differences (between the intercept only model and the fitted model) ≥ 10 were selected. Figure 7 summarises the proportionality of selected model covariates for each location. No one individual ENSO index was consistently or commonly selected, highlighting that the oceanic, atmospheric and coupled ocean-atmospheric interactions that the ENSO indices quantify and their associated interaction and impact on TC activity varies according to sub-region. This demonstrates the benefits of including multiple indices of ENSO that reflect the diversity of the phenomena. Note that the proportionality of individual ENSO indices (light grey bars) cannot be compared with non-ENSO indices (red bars) as only one ENSO index was included in each of the ten independent predictor models (Table S1). However, analysing the proportionality of all ENSO indices (dark grey bars; which can be directly compared with non-ENSO indices) indicates that for five of the eight locations (WNP (28%), China (25%), Hong Kong (25%), Korea (25%) and Vietnam (26%)), ENSO indices are most commonly selected by the automated variable selection procedure. For the Philippines and Thailand, the PMM was the most selected index, accounting for 23% and 20% of indices, respectively. For Japan, the IOD E was the most frequently selected index (18%). For all locations, the DMI was the least commonly selected index, accounting for between < 1% (Hong Kong, Korea and Thailand) and 5% (Japan) of chosen covariates, unlike the individual poles (IOD E and IOD W), which were frequently selected by the automated variable selection procedure.

## Discussion

Seasonal TC behaviour is influenced by multiple climate influences that modulate the conditions suited for cyclogenesis and intensification. Using indices representing these climate influences with an automated covariate selection algorithm, multivariate Poisson regression has been applied to train and test model performance for the WNP basin and seven sub-regions. We tested model performance across nine lead times and found that in many cases, model skill is sufficient to enable skillful predictions up to 6 months before the start of the TY season. This approach has highlighted that for each country, the best performing model varies with lead time and underlines the benefits of applying this flexible and adaptive modelling framework for individual locations within a larger TC basin.

The adaptive modelling framework applied in this analysis facilitates the selection of indices that best captures the variability in TY, STY and TY + STY frequency for the WNP and seven sub-regions. The oceanic and atmospheric response to forcing from climate influences (quantified through climate indices) is not spatially or temporally homogeneous across the entire WNP region. The associated formation and intensification of TCs is sensitive to changes in oceanic and atmospheric conditions^53^, and as such, the selected combination of climate indices varies according to location.

For the WNP, China, Hong Kong, Japan and the Philippines, individual TY and STY outlooks were initialised, trained and validated, both of which demonstrated impressive skill. For Korea, Thailand and Vietnam, a small STY event set meant that individual TY and STY outlooks could not be produced (due to negligible/no model skill), but models for all events (TY + STY; where maximum sustained winds ≥ 64 kt) were initialised, trained and validated for all eight locations considered in this analysis.

Although other statistical^5–11^, dynamical^12–14^ and hybrid statistical–dynamical outlooks^15–17^ have been derived and tested for the WNP region, this study is unique in the following ways.

1. Automated covariate selection determines the optimum combination of input variables (seven indices each with 6-monthly leads—42 covariates in total) for each of the ten predictor models. This is a novel and important component of the modelling. The optimum number and combination of predictors changes between model initialisation periods, locations and for individual TY, STY and TY + STY outlooks, enabling the training and validation of the best possible model.
2. For each location and model initialisation period, ten predictor model outputs are available for analysis. Although this study selected the model with the highest LOOCV SS for further analysis, in theory, all ten model outputs could be used to determine the confidence of the prediction. If this model was used in an operational sense to provide seasonal TY guidance, evaluation of confidence is invaluable.
3. The skill of deriving monthly TY outlooks are tested and show that skillful pre-season outlooks for TYs and STYs (WNP, China, Hong Kong, Japan and Philippines) and all TYs (TY + STY; for all locations considered in this study) can be generated up to 6 months before the start of the season, helping bridge the gap between current sub-seasonal and seasonal climate guidance for the WNP. The skill of in-season outlooks are also tested and are found to provide useful insights into TY, STY and TY + STY frequency for the latter months of the 6-month TY season. Deriving rolling monthly outlooks enables the continual refinement of operational outlooks before and during the season, important given TC behaviour is modulated by changes in ocean and atmosphere predictors.
4. If applied in an ongoing operational sense, the adaptive modelling framework accounts for the most recent changes in TY, STY and TY + STY behaviour, including potential climate change influences and the statistically significant trends observed in the historical record between 1987 and 2020 (Figs. 4 and 6). Our approach enables consideration of the most recent season (i.e. the model is trained considering the most recent TY season), so does not assume stationarity, nor does it use static, historical assumptions about the best combination of predictors to provide a prediction for the next season.
5. The modelling framework used here is computationally inexpensive and can be applied to any user-specified region within the WNP and beyond^28,29^. This approach can also be trained and validated for other sub-regional locations within the WNP (e.g. multiple locations in China), which could form the basis of a future study.

While the modelling framework could technically include any relevant predictor, such as multidecadal, or extra-tropical climate variability, the covariates considered in this analysis were included for practicality, including selecting covariates that are regularly updated 1 month before the month of model initialisation. This is required to generate a prediction. We acknowledge that other indices not included in this analysis may add additional skill to the models; however, these could be considered in future updates to the model. In addition, some of the country-specific domains considered in this analysis are large (e.g. China), and the processes that drive changes in TY and STY events may differ considerably according to location. Future work could apply the modelling framework used here and test it for smaller, regional locations within the WNP region and could also consider the incidence of landfalling TYs and STYs. Also, the objectively defined TY seasons (i.e. the most active 6-month period; Fig. 2), may be longer than they need to be, but as we have shown, using the 6 months captures the majority of TSTD, TY and STY events for each location. Regardless, our models can be applied to user-specified seasons. With time, the addition of more seasons to the event set (currently 34 seasons between 1987 and 2020) will increase the sample size and may improve model skill.

The benefits of skillfully predicting TYs and STYs for specific WNP locations up to 6 months before the start of the TY season are wide-reaching. This modelling has the potential to greatly assist governments, decision-makers, aid agencies, insurers/reinsurers and many others that have to make important decisions under uncertainty. Advance warning up to 6 months before the start of the TY season is particularly useful where above-average TY/STY activity is predicted and may enact a series of decisions that could potentially minimise the loss of life and reduce economic losses. The lead-time provided by the models presented in this study could also be used to underpin weather index insurance and/or parametric insurance solutions for the locations considered.

## Data and model development

### Tropical cyclone data and Outlook regions

This study uses TC best-track data from the International Best-Track Archive for Climate Stewardship (IBTrACSv4)^54^ for the Western North Pacific (WNP; Equator-60° N, 100° E–180°). The following three intensity categories^55^ are applied to IBTrACS: Tropical storm/depression (TSTD) with maximum sustained winds < 64 kt, Typhoon (TY) with maximum sustained winds ≥ 64 kt and < 114 kt (Category 1–3 on the Saffir-Simpson Hurricane Wind Scale) and Super Typhoon (STY) with maximum sustained winds ≥ 114 kt (Category 4 and 5 on the Saffir-Simpson Hurricane Wind Scale). The study period extends between 1987 and 2020. TC data pre-1987 was not considered due to a lack of in situ aircraft reconnaissance before this time^56^, resulting in inconsistent intensity estimates. This analysis does not account for interbasin differences in defining the intensity of TY and STY events and uses the definitions are outlined above.

Regional Areas of Responsibility (AORs) as per the World Meteorological Organizations regional warning areas^57^ are included in this analysis: WNP (0°–60° N, 100° E–180°), Korea (27.5°–45° N, 115°–132.5° E), Hong Kong (30°–10° N, 105°–125° E), Vietnam (variable domain), Thailand (5°–22.5° N, 90°–120° E) and the Philippines (variable domain), many of which overlap (see Fig. 1). The AORs for China (0°–50° N, 95°–175° E) and Japan (0°–60° N, 100° E–180°) are large and overlap a considerable portion of the WNP AOR (0°–60° N, 100° E–180°). Instead, and in order to derive more location-specific guidance for China and Japan, a 500 km buffer was applied to the maximum spatial extents of the coastlines of Mainland China and Japan (Hokkaido prefecture to the north and Okinawa to the south). As such, TCs passing within 500 km of China (15.6°–45.4° N, 103.4°–128.3° E) and Japan (21.5°–50° N, 124.3°–151.7° E) are included in this analysis.

The number of TCs to pass within an AOR is calculated every month, and its maximum intensity within that AOR is recorded and assigned TSTD, TY or STY intensity. The most active 6-month period is used to define the typhoon season. Using this approach, June-November is typhoon season for the WNP, China, Hong Kong, Japan, Philippines, Thailand and Vietnam and for Korea, May–October is the most active 6 month period.

### Predictor variables

Five large-scale modes of ocean–atmosphere variability that are known to drive changes in WNP TC activity are included in this analysis. In total, 16 indices (Table 1) quantify the following modes of variability: ENSO (index 1–10), IOD (index 11–13), PNA (index 14), QBO (index 15) and the PMM (index 16) (see Fig. S1 for a diagrammatic summary). As there is no consensus on which index best represents the ENSO phenomenon^58^, ten unique ENSO indices are included in this analysis (following ^28,29^). However, only one ENSO index at a time is paired with the remaining indices, negating the possibility of multicollinearity^59,60^, resulting in ten individual predictor models (see Table S1).

Each predictor model (seven indices in total) contains six consecutive 1-month values and are dependent on the model initialisation month. For example, for a June-November typhoon season, a lead − 1 outlook is generated in May, 1 month before the start of the season. The predictor model for this lead − 1 outlook contains six consecutive 1-month values starting from April (month 1) extending back for 6 months to November (month 6; the previous year). In total, nine model initialisation months are considered, including six “pre-season” models (lead − 1 to lead − 6) and three “in-season” (lead + 1 to lead + 3) models. Depending on the defined typhoon season, a lead + 1 (lead + 3) outlook is generated in the first (third) month of the season, providing predictions for the remaining five month (3 months) season. For each of the ten predictor models, there are 42 variables (seven indices with 6 monthly values).

### Poisson modelling framework

Following other studies^28,29,61–64^, Poisson regression has been successfully applied to predict historical TC counts, y, using one or more geophysical parameters (predictors). As such, we use multivariate Poisson regression:

1 $$P\left({Y}_{i}=y\right)= \frac{{\mu }_{i}^{y}{\text{exp}}(-{\mu }_{i})}{y!}, y=0, 1, 2, \dots$$

where:

2 $${\mu }_{i}={\text{exp}}(({\beta }_{0}+ \sum_{j}\left({\beta }_{j}{x}_{ij}\right))$$

where μ_*i*_ is the expected number of TC counts with covariate values ${x}_{ij}$ for the $j$ predictors on the $i$th observation. ${\beta }_{j}$ refers to the regression coefficient for each covariate and ${\beta }_{o}$, the intercept. The training period for this analysis is 1987–2020 period (34 TY seasons in total).

Due to the number of variables (42 in total) in each of the ten predictor models, automated variable selection is necessary to select the most skillful combination of predictors while maintaining some degree of parsimony. The stepAIC (Akaike Information Criterion) R function (MASS package^65^) applies a backward and forward stepwise search to select the most appropriate combination of predictors, using the AIC^66^ as a selection criterion for determining when the variable elimination procedure should stop. Poisson regression is then applied using the selected covariates to generate a predicted TC time series. This methodology is applied independently for each of the eight regions, ten predictor models and for all model lead times (nine in total).

The above methodology is initially applied in *training* mode, where the generalised linear model is fitted to the entire time series (1987–2020). The resultant prediction is referred to as the ‘*training*’ prediction. The leave-one-out cross-validation (LOOCV^67^) is also applied using the selected predictors from the variable selection methodology outlined above. Using this approach, the model is trained using n − 1 seasons to produce a hindcast number of events and is iteratively applied in a jackknife fashion to hindcast every historical season in the record^68,69^ (e.g. 51 seasons when applied between 1970 and 2020). This is referred to as the ‘*LOOCV*’ prediction. For both training and LOOCV prediction time series, model skill is tested using a number of criteria including:

1. Pearson correlation coefficient (r; training prediction/LOOCV prediction vs. observed TCs);
2. Mean absolute error (MAE);
3. Skill score (SS): Following^70^, SS is defined as:
  3 $$SS=1- \frac{{MSE}_{f}}{{MSE}_{c}},$$
  where MSE_*f*_ is:
  4 $${\text{MSE}}_{\text{f}}=\frac{1}{{\text{N}}}\sum_{\text{i=1}}^{\text{N}}{\left({\text{f}}_{\text{i}}-{\text{O}}_{\text{i}}\right)}^{2}$$
  where f_*i*_ and O_*i*_ are the *i*th prediction and observation, respectively. Similarly, MSE_c_ is defined by the substitution of climatological values for the predictions, f_i_, in Eq. (4), such that:
  5 $${MSE}_{c}= \frac{1}{N} \sum_{i=1}^{N}{(\overline{O }- {O}_{i})}^{2},$$
  where the climatological prediction is defined by
  6 $$\overline{O }=\frac{1}{N}\sum_{i=1}^{N}{O}_{i}.$$
  A SS of 1.0 (100%) indicates a perfect prediction and negative values indicate predictions that are less accurate than climatology^70^.
4. Strike rate: exact (SR-E), the percentage of seasons where the prediction (training/LOOCV) exactly matches the observation;
5. Strike rate ± 1 (SR ± 1): the percentage of seasons for which the training/LOOCV prediction is ± 1 from the observation.

For each outlook region, model initialisation period and TC category, the skill of each of the ten predictor models are evaluated for both the training and LOOCV predictions. The model with the highest LOOCV SS is selected as the best performing model and skill statistics are reported. The difference in finite-sample corrected AIC (AICc)^66^ between the intercept only and the best performing model is checked to ensure models are not overfitted (AICc values ≥ 10 indicate models are not overfitted).

## Supplementary Information

Supplementary Information.
