# Author Correction: A new approach for location-specific seasonal outlooks of typhoon and super typhoon frequency across the Western North Pacific region

**DOI:** 10.1038/s41598-021-01074-z

**Published:** 2021-10-28

**Authors:** Andrew D. Magee, Anthony S. Kiem, Johnny C. L. Chan

**Affiliations:** 1 Centre for Water, Climate and Land (CWCL), University of Newcastle, Callaghan, Australia; 2 School of Energy and Environment, City University of Hong Kong, Hong Kong, China

Correction to: *Scientific reports* 10.1038/s41598-021-98329-6, published online 30 September 2021

The original version of this Article contained errors.

In the Abstract,

“Using multivariate Poisson regression and considering up to nine modes of ocean-atmospheric variability and teleconnection patterns that influence WNP TC behaviour, thousands of possible predictor model combinations are compared using an automated variable selection procedure.”

now reads:

“Using multivariate Poisson regression and considering up to five modes of ocean-atmospheric variability and teleconnection patterns that influence WNP TC behaviour, thousands of possible predictor model combinations are compared using an automated variable selection procedure.”

Additionally, the Supplementary Information file published with this Article contained an error where the “Southern Annular Mode (SAM)” was incorrectly given in Figure S1. The original Supplementary Information file is provided below.

The original Article and accompanying Supplementary Information file have been corrected.

## Supplementary Information

Supplementary Information.

