# Supplementary material for: A new approach for location-specific seasonal outlooks of typhoon and super typhoon frequency across the Western North Pacific region
Source: Sci Rep. 2021 Sep 30;11:19439. doi: 10.1038/s41598-021-98329-6 (PMC8484678; doi:10.1038/s41598-021-98329-6)
Supplement: Supplementary file 1 — Supplementary Information. [file 41598_2021_98329_MOESM1_ESM.docx]

**A new approach for location-specific seasonal outlooks of typhoon and super typhoon frequency across the Western North Pacific region**

Andrew D. Magee^1*^, Anthony S. Kiem^1^, Johnny C. L. Chan^2^

^1^ Centre for Water, Climate and Land (CWCL), University of Newcastle, Australia

^2^ School of Energy and Environment, City University of Hong Kong, Hong Kong

*Corresponding author: [andrew.magee@newcastle.edu.au](mailto:andrew.magee@newcastle.edu.au)

**Supplementary Material**

*Submit to: Scientific Reports*

**Table S1:** Ten predictor models used in this analysis. Individual ENSO indices are combined with other indices including the IOD E, IOD W, DMI, PNA, QBO and PMM. See Figure S1 for a diagram illustrating the indices considered in this analysis and Table 1 for more details on indices.

| **Model** | **ENSO index** | **Other indices** |
| --- | --- | --- |
| 1 | NINO1+2 | IOD E  IOD W  DMI  PNA  QBO  PMM (SST only) |
| 2 | NINO3 |  |
| 3 | NINO3.4 |  |
| 4 | NINO4 |  |
| 5 | Southern Oscillation Index (SOI) |  |
| 6 | Coupled ENSO Index (CEI) |  |
| 7 | Oceanic NINO Index (ONI) |  |
| 8 | Trans Nino Index (TNI) |  |
| 9 | ENSO Modoki Index (EMI) |  |
| 10 | ENSO Longitude Index (ELI) |  |


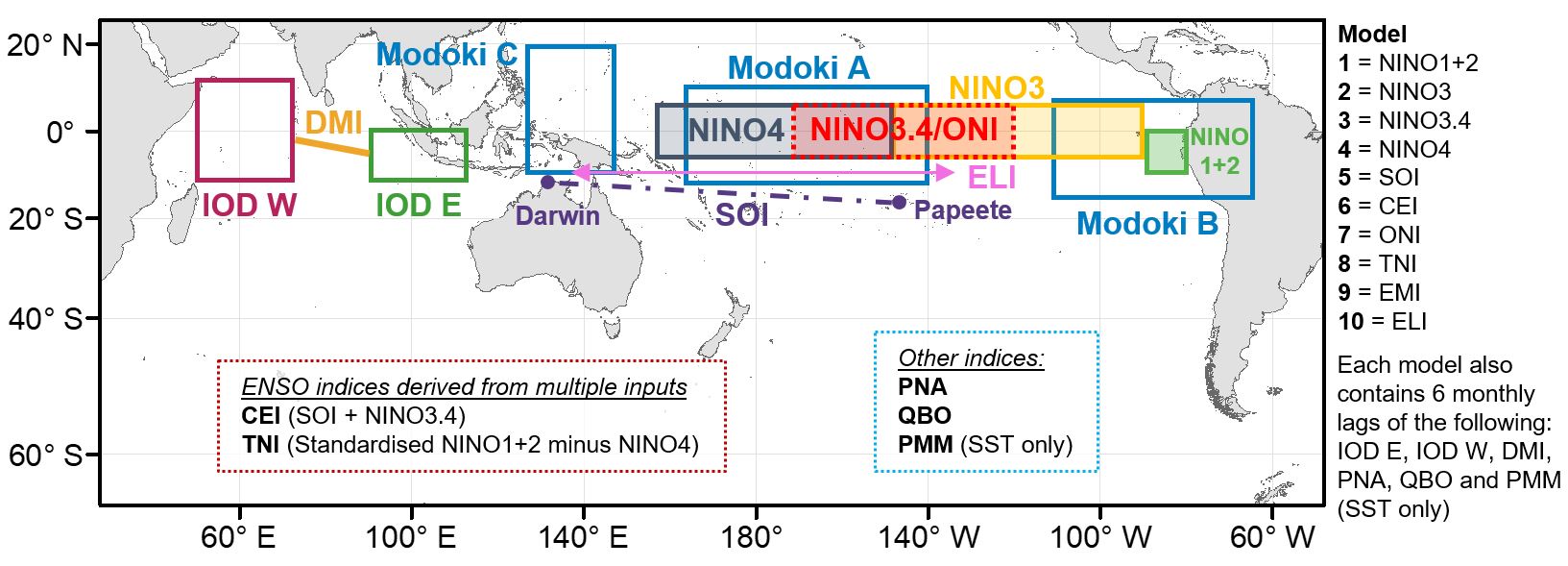


**Figure S1:** Climate indices (model covariates) considered in this analysis. Indices representing ENSO include: NINO1+2, NINO3, NINO4, NINO3.4, SOI, EMI, CEI, ONI, TNI and the ELI. Other indices include IOD W, IOD E, DMI, PNA, QBO and PMM (the index acronyms are defined in Table 1). Ten predictor model combinations used in this analysis are summarised to the right of the panel and in Table 1. Six monthly leads are generated for each outlook initiation period (lead-6 to lead+3).


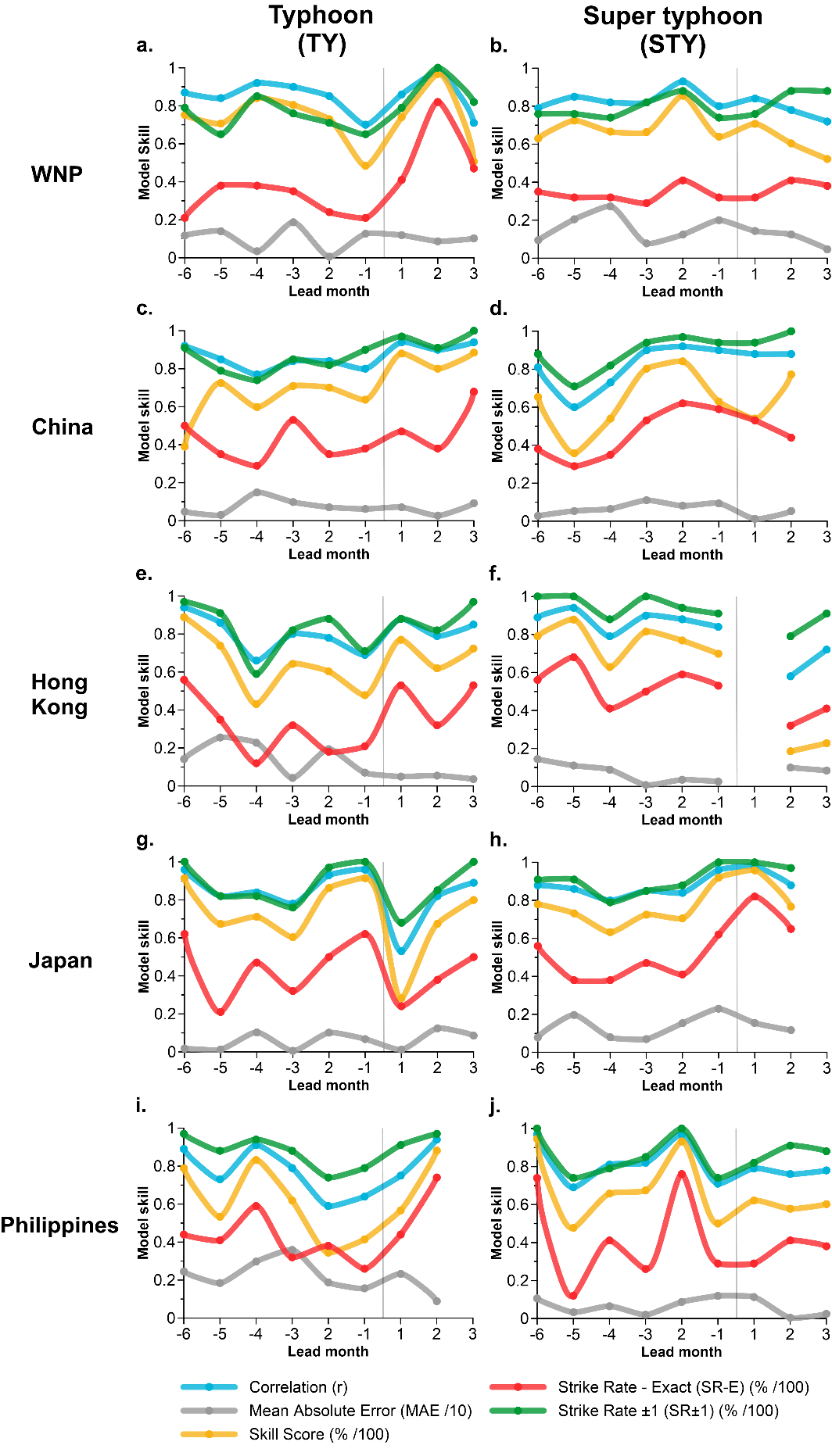


**Figure S2:** Evaluation of training model performance for model lead times (leads-6 to +3) for TYs (left panels) and STYs (right panels) for the WNP (panels a-b), China (panels c-d), Hong Kong (panels e-f), Japan (panels g-h) and the Philippines (panels i-j) for each respective six month typhoon season between 1987 and 2020. The vertical line separates pre-season (lead-1 to lead-6) and in-season (lead+1 to lead+3) outlooks. Lead months with missing model performance statistics indicates negligible/no model skill.


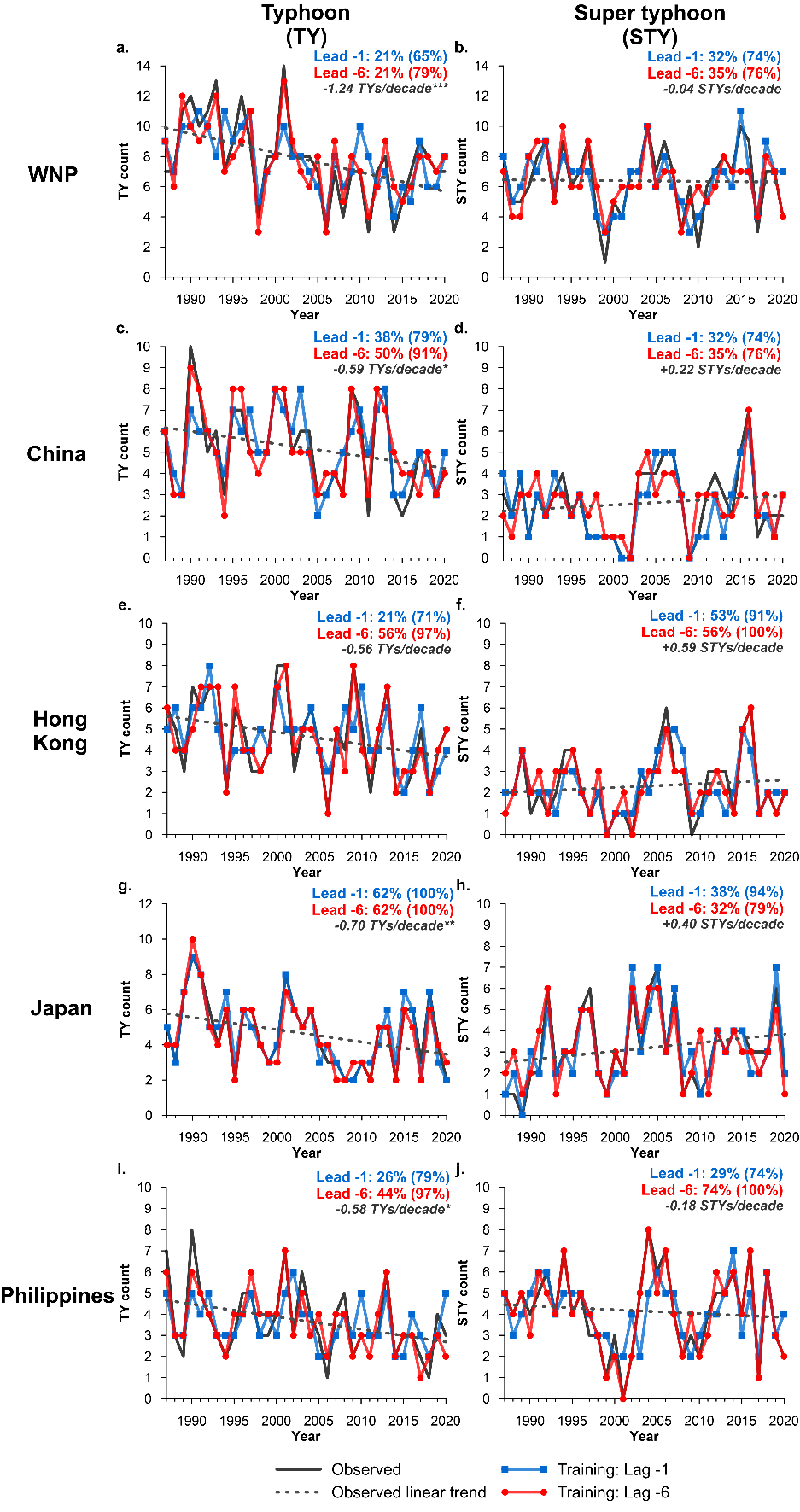


**Figure S3:** Comparison of observed and predicted TYs (left column) and STYs (right column) for the NWP (panels a-b), China (panels c-d), Hong Kong (panels e-f), Japan (panels g-h) and the Philippines (panels i-j) between 1987 and 2020. The training prediction is compared for two pre-season periods: lead-1 (one month before the start of the typhoon season; blue line) and lead-6 (six months before the start of the typhoon season; red line). On-panel percentage values indicate training model SR-E (SR+-1 in parentheses) for models lead-1 and lead-6. Dashed line represents observed linear trend with on-panel trend (/decade) summarised in grey italics with statistical significance (Mann-Kendall test) denoted by an asterisk (* = significant at 90%; ** = significant at 95%; *** = significant at 99%).


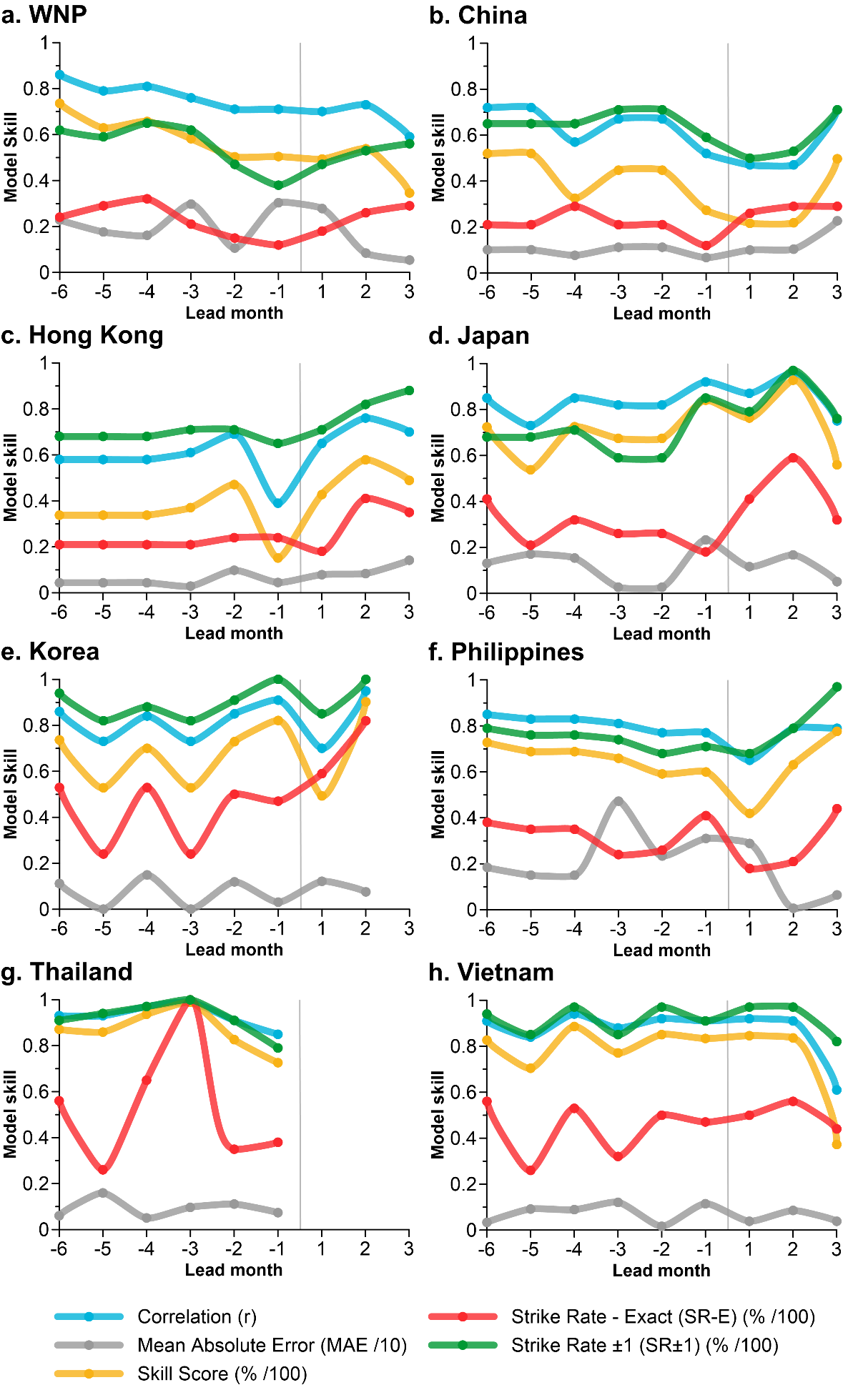


**Figure S4:** Evaluation of training model performance for all typhoons (TY and STY events) for eight locations between 1987 and 2020. The vertical line separates the pre-season (lead-1 to lead-6) and in-season (lead+1 to +3) outlooks.


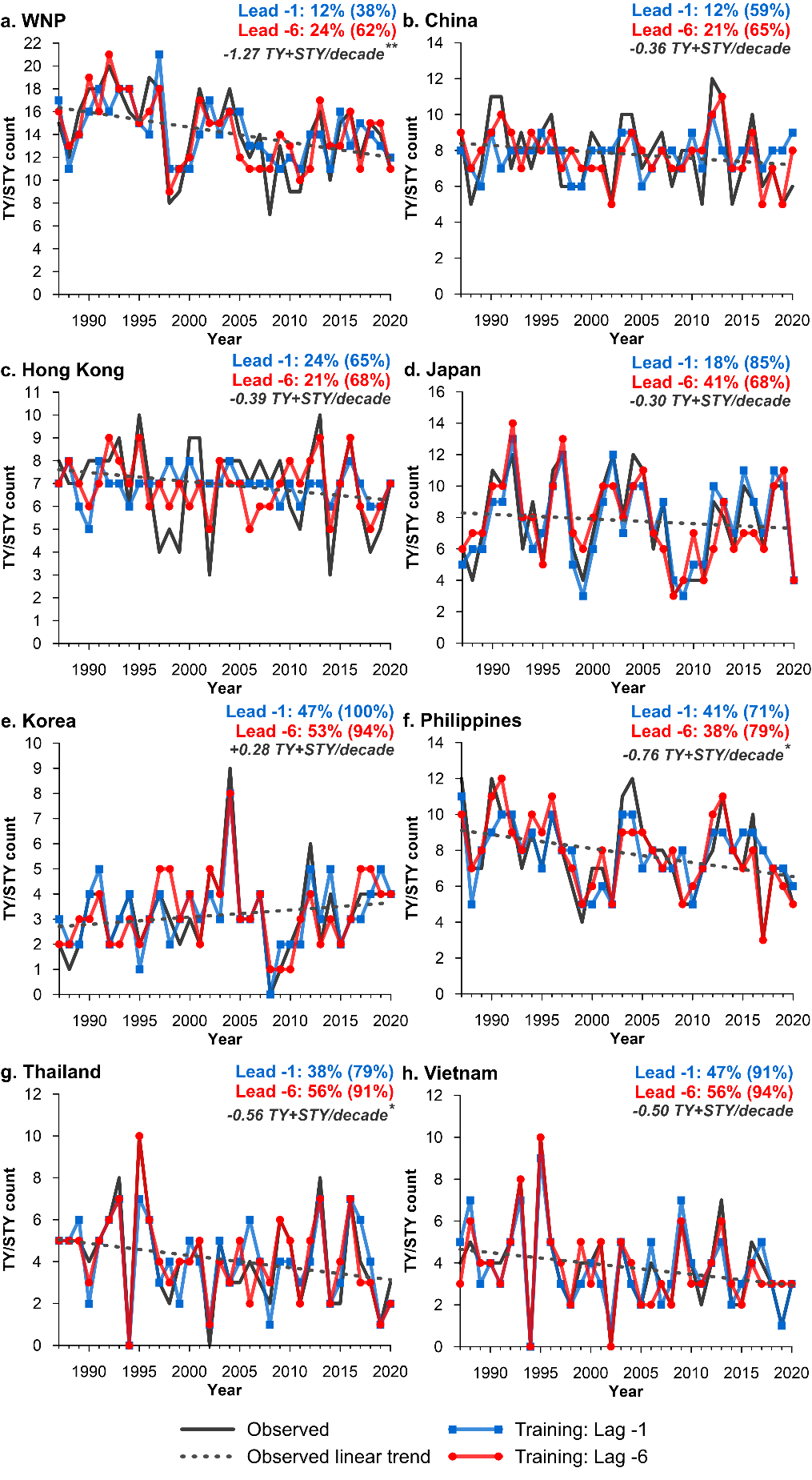


**Figure S5:** Comparison of observed and predicted typhoons (all typhoons including TY and STY) between 1987 and 2020. The training model prediction is compared for two pre-season periods: lead-1 (one month before the start of the typhoon season; blue line) and lead-6 (six months before the start of the typhoon season; red line). On-panel percentage values indicate predictor model SR-E (SR+-1 in parentheses) for models lead-1 and lead-6. Dashed line represents observed linear trend with on-panel trend (/decade) summarised in grey italics with statistical significance (Mann-Kendall test) denoted by an asterisk (* = significant at 90%; ** = significant at 95%; *** = significant at 99%).
